# Should Nurses Strike?

**Published:** 1921-04-16

**Authors:** Millicent I. Wade


*To the Editor of* The Hospital.

### Should Nurses Strike?

*To the Editor of* The Hospital.

Sir,—"Ierne's" diverting question respecting the desirability of our smoking—nothing stronger than a cigarette, I suppose?—might easily be followed with the more serious query, "Should nurses strike?"

The old order changeth, giving place to—well—"She's only a nurse from a Labour Exchange, my dear!" Just as the present is fraught with peril, so is the future pregnant with possibilities. A few years ago, if it were considered that we required shepherding, the selectness of the flock was not disputed, and even to-day nurses have no inclination to parade the streets, with the friendly assistance of mounted police, when out of appointment. They have other less strenuous ways of raising and augmenting private means.

Nurses are beginning to consider, however, that their inclusion in the National Unemployment scheme was merely an afterthought, typical of the blundering, unimaginative, official mind, and to strongly resent this arrangement on the part of the authorities, which savours of prompting how they shall spend their own cash. It is disquieting, very undignified, and uncomfortable for a nurse to feel as if her one leg only is in hospital, but she has a crutch at the nearest Labour Exchange.

Of course, we are now amenable, also, to that vexatious regulation which makes it possible for a person receiving unemployment benefit to be sent any distance away from home towns, when and wherever a vacancy occurs in the United Kingdom. To refuse is to forfeit all claims to which an employable person is entitled when out of employment. In some instances it is proposed to deduct in a lump sum from our next month's engagement money the contributions payable since the Unemployment Act came in force. Naturally, no question regarding the convenience or otherwise of this impolitic mulcting will arise. It must be an especial regret that the State finger having written, we should find in its message the implication: what was once considered to be the joy of attending the afflicted is fast becoming the business of healing the sick.

Yes, I think this endeavour to place us on almost the same business footing as industrial workers has doubtless assisted in bringing a strike of nurses within measurable distance.—Yours truly,

Millicent I. Wade.

